# Biochemical responses of rice roots to cold stress

**DOI:** 10.1186/s40529-019-0262-1

**Published:** 2019-07-12

**Authors:** Ching Hsin Hsu, Yi Ting Hsu

**Affiliations:** Department of Agronomy, National Chung Hsing University, Taichung, Taiwan, ROC

**Keywords:** Cold responses, Rice seedling, Root, Oxidative stress

## Abstract

**Background:**

Cold stress is the main factor that reduces rice yield in subtropical areas, especially at the seedling stage. Most of the current studies on cold stress focus the responses of rice shoots to cold stress. Limited studies are available on that of rice roots to cold stress. This study aimed to illustrate the biochemical responses of rice root under cold treatment, and subject to the establishment of cold stress-related biochemical traits for rice breeding or cropping-adjustment.

**Results:**

Our results showed that the growth of rice seedling diminished under cold stress with difference extents among eight rice cultivars of most productive in Taiwan. Under cold treatments, the tested cultivars with higher growth rate had a higher level of hydrogen peroxide (H_2_O_2_) in the shoots but had a lower level in the roots. In contrast, the tested cultivates with low growth rate had higher levels of H_2_O_2_ in the roots but a lower level in the shoots. Meanwhile, higher MDA contents and higher cell-damage related electrolyte leakage were also found in the roots not in the shoots, suggesting that cold stress might induce oxidative stress in the roots, not in the shoots. Furthermore, the activity analysis of four antioxidant enzymes, namely superoxide dismutase (SOD), catalase (CAT), ascorbic peroxidase (APX), and glutathione reductase (GR), revealed that cold stress could increase SOD and CAT activities in the roots.

**Conclusions:**

In summary, low H_2_O_2_ and low MDA contents along with lower SOD and CAT activities in rice root could be the biochemical traits of cold responses in rice seedlings. The results are hoping to have a contribution to the rice breeding or cropping-adjustment on cold tolerance.

**Electronic supplementary material:**

The online version of this article (10.1186/s40529-019-0262-1) contains supplementary material, which is available to authorized users.

## Background

Rice (*Oryza sativa* L.) is mainly cultivated in tropical and subtropical regions and provides a substantial food resource. Because of climate change and an increase in extreme temperatures, the yield of rice has gradually declined (Solomon et al. 2007). The incidence of low temperature is one factor responsible for the declining yield, especially at the seedling stage (Aghaee et al. 2011; Bhattacharjee 2013; Dashtmian et al. 2014). This cost of cold damage is approximately 75% of all disaster loss in Taiwan (Additional file 1: Figure S1).

In general, cold temperatures of 0–15 °C can reduce the crop survival rate, inhibit photosynthesis, retard growth, and block the synthesis of proteins, lipids, and carbohydrates (Setter and Greenway 1988; Aghaee et al. 2011; Liu et al. 2013). At the seedling stage, rice is more sensitive to low temperatures because low temperatures can inhibit seed germination (Morsy et al. 2006; Baruah et al. 2009) and also retard seedling growth, resulting in leaf curving, shoot shortening, and few tillers (Dashtmian et al. 2014). In addition, low temperatures may cause the accumulation of reactive oxygen species (ROS), such as superoxide anion, singlet oxygen, and hydrogen peroxide (H_2_O_2_), which leads to lipid peroxidation, electrolyte leakage, and membrane damage (Kuk et al. 2003; Hung et al. 2008; Bhattacharjee 2013).

Cold temperatures have been found to damage the rice root tissue, resulting in a decrease in water obtained by the roots and upward nutrient transport to the shoot, retarding the growth of rice seedlings (Setter and Greenway 1988; Neilson et al. 2013). However, the biochemical mechanism is not clear, especially with regard to the roles of ROS. Nevertheless, the study of salt stress on rice root by Lin and Kao (2001a) revealed an increase in ionically bound cell-wall peroxidase activity after NaCl treatment in rice roots, which resulted in H_2_O_2_ generation and thus inhibited the growth of rice roots. Lin and Kao (1999, 2001a) have found that NaCl treatment inhibited the root growth of rice seedlings and agreed with the theory of H_2_O_2_ induced cell-wall stiffening process (Fry 1986; Lin and Kao 2001b). We found that Cd toxicity in rice leaves is due to H_2_O_2_ accumulation (Hsu and Kao 2007) and further reported that H_2_O_2_ accumulation is responsible for Cd-inhibited root growth of rice seedlings (Cho et al. 2012), where Cd could inhibit the activity of catalase (CAT), which is supposed to break down H_2_O_2_ into water and oxygen in rice roots. Thus, Cho et al. (2012) suggested that a decrease in CAT may result in the accumulation of H_2_O_2_ in the rice root. In the root cell of *Arabidopsis*, H_2_O_2_ was also found to be involved in the nutrient-deficiency response (Shin and Schachtman 2004; Shin et al. 2005), and it might play a role in the sensing and signaling of N, P, K, and S nutrients (Schachtman and Shin 2007).

Therefore, our study was attempting to analyze mechanisms underlying the responses of the rice root to cold stress, especially with regard to the oxidative status of the root and the activities of four antioxidant enzymes, namely superoxide dismutase (SOD), CAT, ascorbate peroxidase (APX), and glutathione reductase (GR). The accumulation of ROS is the beginning of oxidative stress and results in the lipid peroxidation of the cell membrane, which can be expressed by the increase of malondialdehyde (MDA) contents (Hung et al. 2008; Bhattacharjee 2013). In respond to oxidative stress, plant tissue will increase the activity of SOD to reduce the ROS level and generate H_2_O_2_. Since H_2_O_2_ is toxic to the cell, the activities of CAT or APX will be strengthened to decrease H_2_O_2_ contents (Chao et al. 2010; Chou et al. 2012). The activity of APX can be maintained in couple with the action of GR. These biochemical traits may be used as selecting marker for a rice breeding project or cropping-adjustment in cold tolerance. This study aimed to illustrate the link between rice root responses and cold stress by analyzing these biochemical traits on eight rice cultivars.

## Methods

The most productive cultivars of rice (*Oryza sativa* L.) in Taiwan are these eight, namely Taitung 30 (TT30), Tainan 11 (TN11), Tainung 71 (TNG 71), Kaohsiung 139 (KH139), Tai-Keng 16 (TK16), Tai-Keng 9 (TK9), Tai-Keng 14 (TK14), and Taichung-Sen 10 (TCS10). This study selected these eight cultivars for cold treatment. Rice seeds were kept in an incubator at 37 °C for 48 h to break seed dormancy. Twelve sprouts of the same size from one cultivate were selected and placed in a covered plate with a 9-cm wet filter paper as one replicate. The plates were kept in a growth chamber at a light/dark cycle of 14/10 h with a light intensity of 200 μmole photons/m^2^/s. The temperature of the growth chamber was set at 15 °C for cold treatment and 27 °C for control. After 4 days of treatment, the chamber temperature was adjusted to 27 °C, and plates were kept uncovered for 3 days and watered every day before harvesting for growth analysis. Each cultivates had four replicates in one experiment. Data from three experiments were collected for statistical analysis. From the 12 sprouts of each replicate, nine seedlings in the middle range of length were collected for the measurement of length, fresh weight, and dry weight of the shoot and root.

Protein and chlorophyll contents were determined according to the methods of Bradford (1976) and Wintermans and de Mots (1965), respectively. The degree of lipid peroxidation was expressed as the content of malondialdehyde (MDA), which was determined using the method reported by Health and Packer (1968). The tissue content of H_2_O_2_ was determined using the method reported by Jana and Choudhuri (1982), which has previously been used for rice seedlings (Lin and Kao 2001a, b; Hsu and Kao 2004). The method for measuring electrolyte leakage was modified from that used by Blum and Ebercon (1981) and Dashtmian et al. (2014). The detached roots were soaked in 10 mL of deionized water for 16 h for the first water conductivity measurement (C1) and then soaked in boiling water for 1 h for the second measurement (C2). The degree of electrolyte leakage was calculated as C1/C2 × 100.

The activities of SOD, CAT, APX, and GR were determined according to methods that have been tested in rice seedlings (Chou et al. 2012; Chao et al. 2010). SOD was determined according to the method used by Paoletti et al. (1986). One unit of SOD was defined as the amount of enzyme that inhibits the rate of NADH oxidation observed in the blank by 50%. The CAT activity was assayed by measuring the initial rate of disappearance of H_2_O_2_ (Kato and Shimizu 1985). APX was determined according to the methods used by Nakano and Asada (1981). A decrease in the ascorbate concentration was correlated to a decrease in optical density at 290 nm. GR was determined using the method reported by Foster and Hess (1980). One unit of GR was defined as the amount of an enzyme that reduces 1 absorbance of reading on 340 nm/min. Activities of all enzymes expressed on the basis of fresh weight.

Statistical analyses, including standard error, analysis of variance, least significant difference multiple comparisons, *t*-test, and regression test, were performed using the Statistical Analysis System (SAS 9.4).

## Results

### Cold stress inhibited the growth of rice seedlings

Eight of the most productive rice cultivars in Taiwan were chosen for cold treatments (Additional file 1: Table S1). According to the pedigree of these cultivars, the ratio of their genome origin was estimated and displayed in Additional file 1: Table S1. In general, most of them contain a high portion of *Japonica* type genome and less portion of *Indica* type genome. It was noticed that two cultivars (TN11 and KH139) are 100% from *Japonica* genotype, and one cultivar (TCS10) is 100% from *Indica* genotype. The rice origin of *Japonica* genotype is from the temporal region and that of *Indica* genotype is from subtropical and tropical regions. The native rice of Taiwan is *Indica* type. The current *Japonica* type of rice is introduced from Japan. Thus it was general to be believed that rice of *Japonica* genotype is more tolerant to cold stress than that of *Indica* genotype. However, the initial breeding project of Taiwan was focused on heat tolerance and, as a result, more and more new *Japonica* type rice is able to grow in the south of Taiwan. Less attention had been put on cold tolerant. Thus, even though the current popular rice varieties in Taiwan are *Japonica* type, Taiwan is still in the suffering of yield loss of rice by cold stress (Additional file 1: Figure S1). We speculated that some of the cold stress-related genomes might be lost during previous breeding selection. Thus, even with a high portion of *Japonica* type genome in most productive eight rice cultivars, the cost of cold damage was as high as 75% of all disaster loss in the subtropic region of Taiwan. To establish biochemical traits of cold stress-related would be valuable for future rice breeding on cold tolerance. The results in Additional file 1: Figure S1 also showed January and February are the 2 months of having the most loss on cost, during which the rice is on the stage of the seedling. Thus, this study focused on the performance of rice seedlings under cold stress.

**Fig. 1:**
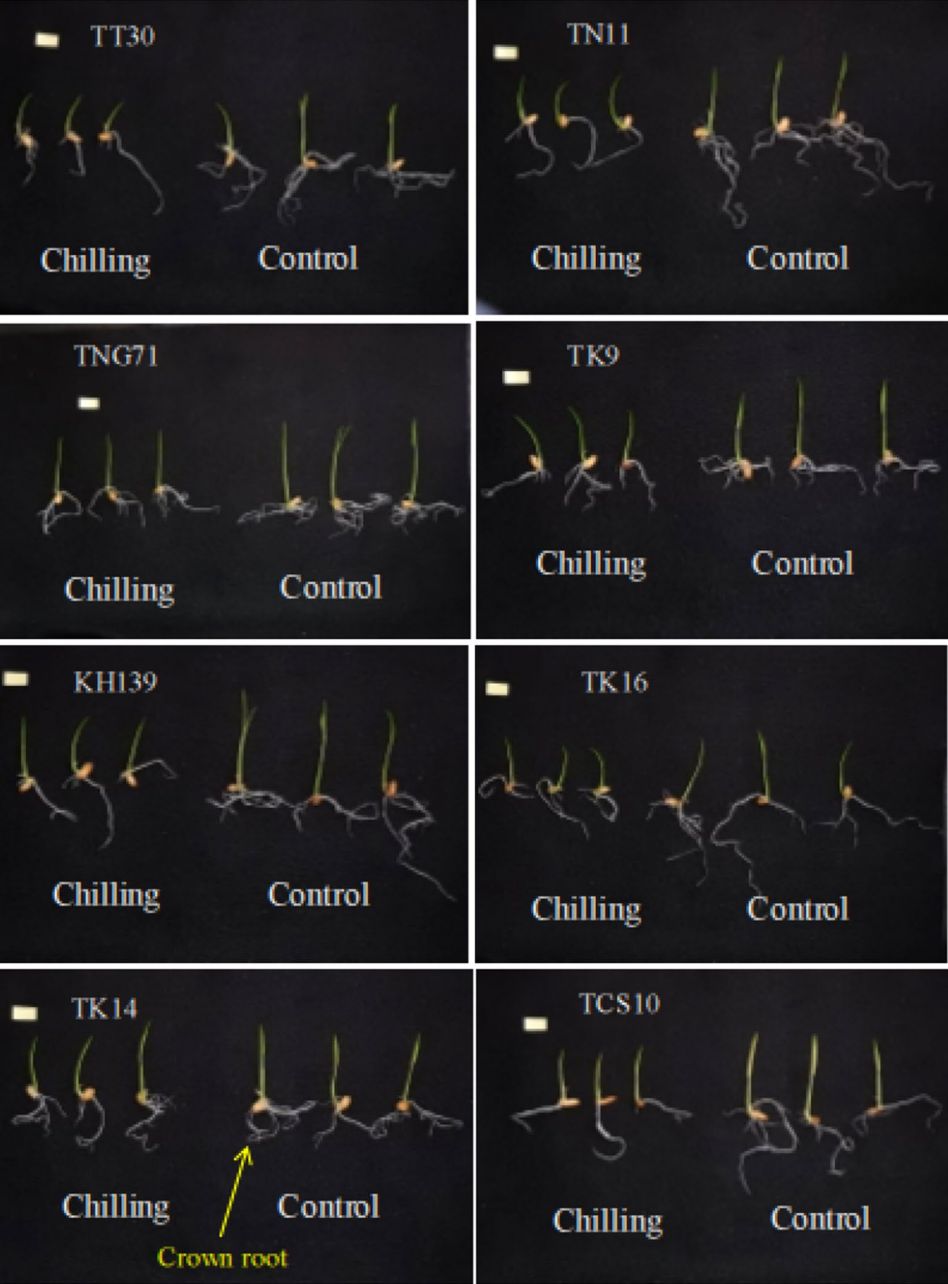
The cold stress response of rice seedlings. The temperature was set at 15 °C for cold treatment and 27 °C for control. After 4 days of treatment, the temperature was adjusted to 27 °C for 3 days before harvesting. Bar = 1 cm

The rice seedlings of eight cultivars were treated with a cold temperature at 15 °C for 4 days that is the frequent duration of cold stress in Taiwan. After the cold treatment, the seedlings were re-warmed at 27 °C for 3 days and followed with harvesting and growth analysis. The treatment of 27 °C to 27 °C for 7 days was considered as the control. The results of cold treatment showed that the growth of shoots decreased in all testing cultivars (Fig. 1) as compared with the control in regarding the fresh weight, dry weight, and length (Table 1). The results were similar to that of roots (Table 2 and Fig. 1). Moreover, the variance analysis on shoot growth (Table 1) demonstrated that the extents of growth were significantly different within genotypes and between treatments, meanwhile the interactions of variance on genotypes and cold treatment (G × T) were also differing significantly. Similar to the results obtained for the shoot, the extents of root growth were differing significantly within genotypes and between treatments, and also to the interactions of variance on genotypes and cold treatment (G × T). In addition, the results of Table 1 showed that cultivar TCS10 (100% *Indica* genotype) was the most affected by cold treatment, with a 41.6% reduction on shoot length. Among the other seven cultivars which contain more or less of *Japonica* type genome, cultivar TK14 (12.5% *Indica* genotype) was the least affected by cold treatment with a 10.6% reduction on shoot length. In Table 2, the root length of cultivar TCS10 (100% *Indica* genotype) was the least affected by cold treatment, with a 24% reduction in root length. The next was cultivar TK9 (50% *Indica* genotype) with a 28.4% reduction in root length. Cultivar TT30 (93.75% *Japonica* genotype) was the most affected by cold treatment with 51.3% reduction in root length. The next two cultivars of vulnerable to cold treatment were TN11 and KH139, both were 100% *Japonica* genotype, Thus, it was likely that the more of portion containing with *Japonica* type genome in testing cultivars, the less of shoot length reduction corresponding to cold stress. In opposition with the results in shoot length, the more of portion containing with *Japonica* genotype genome in testing cultivars, the more of root length reduction corresponding to cold stress. Seemingly, the cultivars mainly with a high portion of *Japonica* genotype genome might keep their shoot to grow under cold stress. The cultivars mainly with a high portion of *Indica* genotype genome might keep their root to grow under cold stress.

**Table 1:** The cold stress response of rice seedling in the shoot

Genotype	Temperature (°C)	Fresh weight (g)	Dry weight (g)	Height (cm)	Height reduction (%)
TT30	27 → 27 15 → 27	^#^0.101^bc^ 0.071^fg^	0.0184^b^ 0.0093^ef^	3.22^def^ 2.53^ghij^	21.5
TN11	27 → 27 15 → 27	0.077^ef^ 0.055^h^	0.0139^c^ 0.0068^g^	3.57^cd^ 2.12^ij^	30.6
TNG71	27 → 27 15 → 27	0.120^a^ 0.068^fg^	0.0226^a^ 0.0123^cd^	4.82^a^ 3.45^de^	28.4
TK9	27 → 27 15 → 27	0.099^cd^ 0.064^gh^	0.0190^b^ 0.0106^de^	3.50^de^ 2.67^ghi^	23.7
KH139	27 → 27 15 → 27	0.103^bc^ 0.066^fgh^	0.0192^b^ 0.0078^fg^	4.00^bc^ 2.85^fgh^	28.7
TK16	27 → 27 15 → 27	0.088^de^ 0.055^h^	0.0140^c^ 0.0065^g^	3.35^de^ 2.35^ij^	29.1
TK14	27 → 27 15 → 27	0.096^cd^ 0.063^gh^	0.0183^b^ 0.0073^fg^	3.02^defg^ 2.70^ghi^	10.6
TCS10	27 → 27 15 → 27	0.114^ab^ 0.053^h^	0.0201^b^ 0.0066^g^	4.25^b^ 2.48^hij^	41.6
^$^ANOVA analysis					
Genotype		**	**	**	
Temperature		**	**	**	
G × T		**	**	**	

The temperature was set at 15 °C for cold treatment and 27 °C for control. After 4 days of treatment, the temperature was adjusted to 27 °C for 3 days before harvesting

^#^Different letters represent the significant difference with LSD tests among cultivars or treatments (p < 0.05)

^$^G × T: effect of genotype and temperature interaction; *p < 0.05; **p < 0.01

**Table 2:** The cold stress response of rice seedling in the root

Genotype	Temperature (°C)	Fresh weight (g)	Dry weight (g)	Length (cm)	Length reduction (%)
TT30	27 → 27 15 → 27	^#^0.211^b^ 0.107^fg^	0.0316^a^ 0.0143^ef^	8.12^a^ 3.95^e^	51.3
TN11	27 → 27 15 → 27	0.134^e^ 0.070^h^	0.0206^d^ 0.0083^h^	6.68^b^ 4.43^de^	33.7
TNG71	27 → 27 15 → 27	0.246^a^ 0.121^ef^	0.0344^a^ 0.0156^e^	8.22^a^ 5.57^c^	32.2
TK9	27 → 27 15 → 27	0.182^cd^ 0.094^g^	0.0240^bc^ 0.0118^fg^	6.63^b^ 4.75^cde^	28.4
KH139	27 → 27 15 → 27	0.196^bc^ 0.088^gh^	0.0254^bc^ 0.0097^gh^	8.03^a^ 4.53^de^	43.6
TK16	27 → 27 15 → 27	0.182^cd^ 0.083^gh^	0.0266^b^ 0.0116^fg^	7.97^a^ 5.45^c^	31.6
TK14	27 → 27 15 → 27	0.169^d^ 0.092^gh^	0.0241^bc^ 0.0112^fgh^	6.58^b^ 4.60^de^	30.1
TCS10	27 → 27 15 → 27	0.161^d^ 0.098^fg^	0.0229^cd^ 0.0139^ef^	6.82^b^ 5.18^cd^	24.0
^$^ANOVA analysis					
Genotype		**	**	**	
Temperature		**	**	**	
G × T		**	**	**	

The temperature was set at 15 °C for cold treatment and 27 °C for control. After 4 days of treatment, the temperature was adjusted to 27 °C for 3 days before harvesting

^#^Different letters represent the significant difference with LSD tests among cultivars or treatments (p < 0.05)

^$^G × T: effect of genotype and temperature interaction; *p < 0.05; **p < 0.01

### Cold stress affect the contents of the protein and chlorophyll

**Fig. 2:**
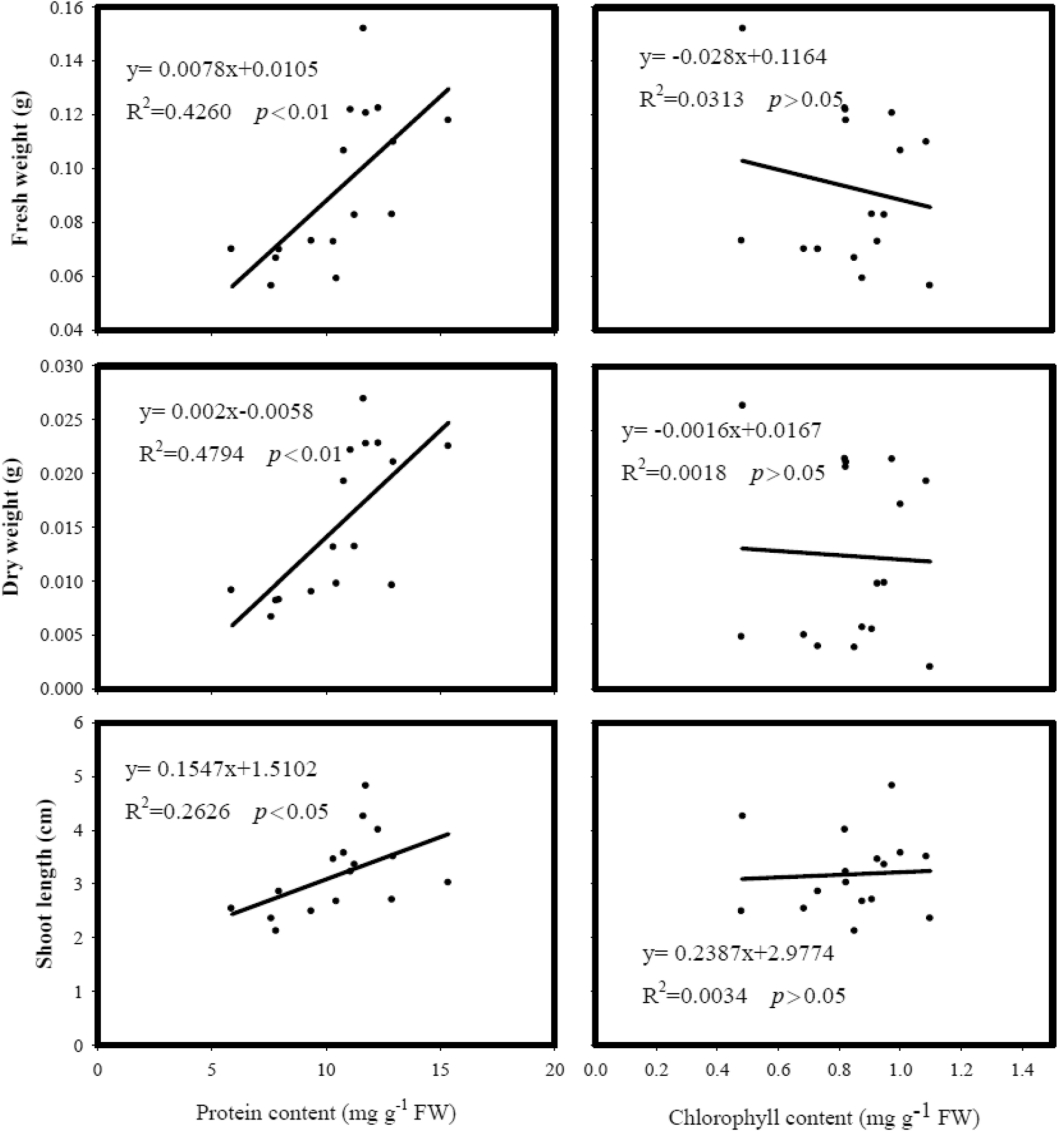
The relationships of shoot protein contents and of chlorophyll contents with rice seedling fresh weight, dry weight and length under cold treatment among eight cultivars. The temperature was set at 15 °C for cold treatment and 27 °C for control. After 4 days of treatment, the temperature was adjusted to 27 °C for 3 days before harvesting

The results of Table 3 showed a decrease in protein content in both roots and shoots of eight cultivars after cold treatment. Additionally, the results of the regression analysis (Fig. 2) revealed that the protein content was positively correlated with shoot growth including fresh weight, dry weight, and length, suggesting the more of protein content, the more of shoot growth. On the contrary, the results in Table 3 showed that the chlorophyll contents were not affected in most cultivars. In addition, the results of the regression analysis (Fig. 2) revealed that the chlorophyll contents were not correlated with shoot growth among all tested cultivars. Therefore, the results of the above suggested that the rice chlorophyll content was less sensitive to cold stress than the protein content (Table 3).

**Table 3:** The cold stress response of rice seedlings

Genotype	Temperature (°C)	Root	Shoot	
		Protein content (mg/gFW)	Protein content (mg/g FW)	Chlorophyll content (mg/g FW)
TT30	27 → 27 15 → 27	^#^6.36^e^ 11.16^bcd^	11.1^cdef^ 5.9^h^	0.82^bcd^ 0.69^d^
TN11	27 → 27 15 → 27	5.98^e^ 10.45^cd^	10.8^def^ 7.9^g^	1.00^ab^ 0.85^bcd^
TNG71	27 → 27 15 → 27	6.86^e^ 10.56^cd^	11.8^bcde^ 10.3^ef^	0.97^ab^ 0.93^abc^
TK9	27 → 27 15 → 27	7.52^e^ 11.69^abc^	13.0^b^ 10.5^ef^	1.09^a^ 0.88^bcd^
KH139	27 → 27 15 → 27	6.34^e^ 10.62^cd^	12.3^bcd^ 8.0^g^	0.82^bcd^ 0.73^cd^
TK16	27 → 27 15 → 27	6.92^e^ 12.46^ab^	11.3^bcde^ 7.6^gh^	0.95^ab^ 1.10^a^
TK14	27 → 27 15 → 27	9.71^d^ 12.92^a^	15.4^a^ 12.9^bc^	0.82^bcd^ 0.91^abc^
TCS10	27 → 27 15 → 27	6.73^e^ 11.65^abc^	11.7^bcde^ 9.4^fg^	0.49^e^ 0.48^e^
^$^ANOVA test				
Genotype		**	**	**
Temperature		**	**	ns
G × T		ns	ns	ns

The temperature was set at 15 °C for cold treatment and 27 °C for control. After 4 days of treatment, the temperature was adjusted to 27 °C for 3 days before harvesting

*ns* non-significant

^#^Different letters represent the significant difference with LSD tests among cultivars or treatments (p < 0.05)

^$^G × T: effect of genotype and temperature interaction; *p < 0.05; **p < 0.01

### Cold stress-induced oxidative stress on the root

**Fig. 3:**
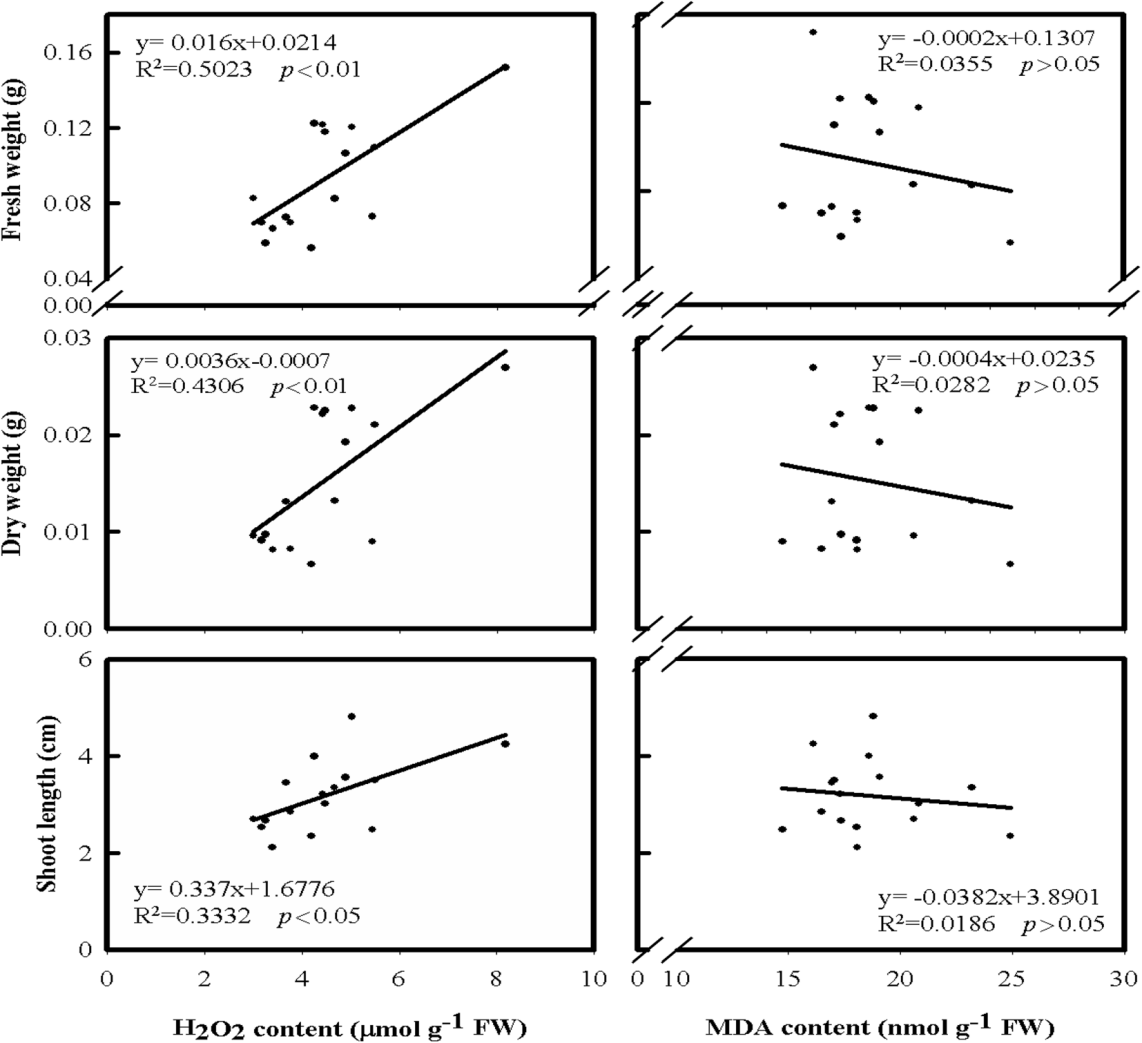
The relationships of leaf H_2_O_2_ and MDA contents with shoot fresh weight, dry weight and length under cold treatment among eight rice cultivates. The temperature was set at 15 °C for cold treatment and 27 °C for control. After 4 days of treatment, the temperature was adjusted to 27 °C for 3 days before harvesting

**Fig. 4:**
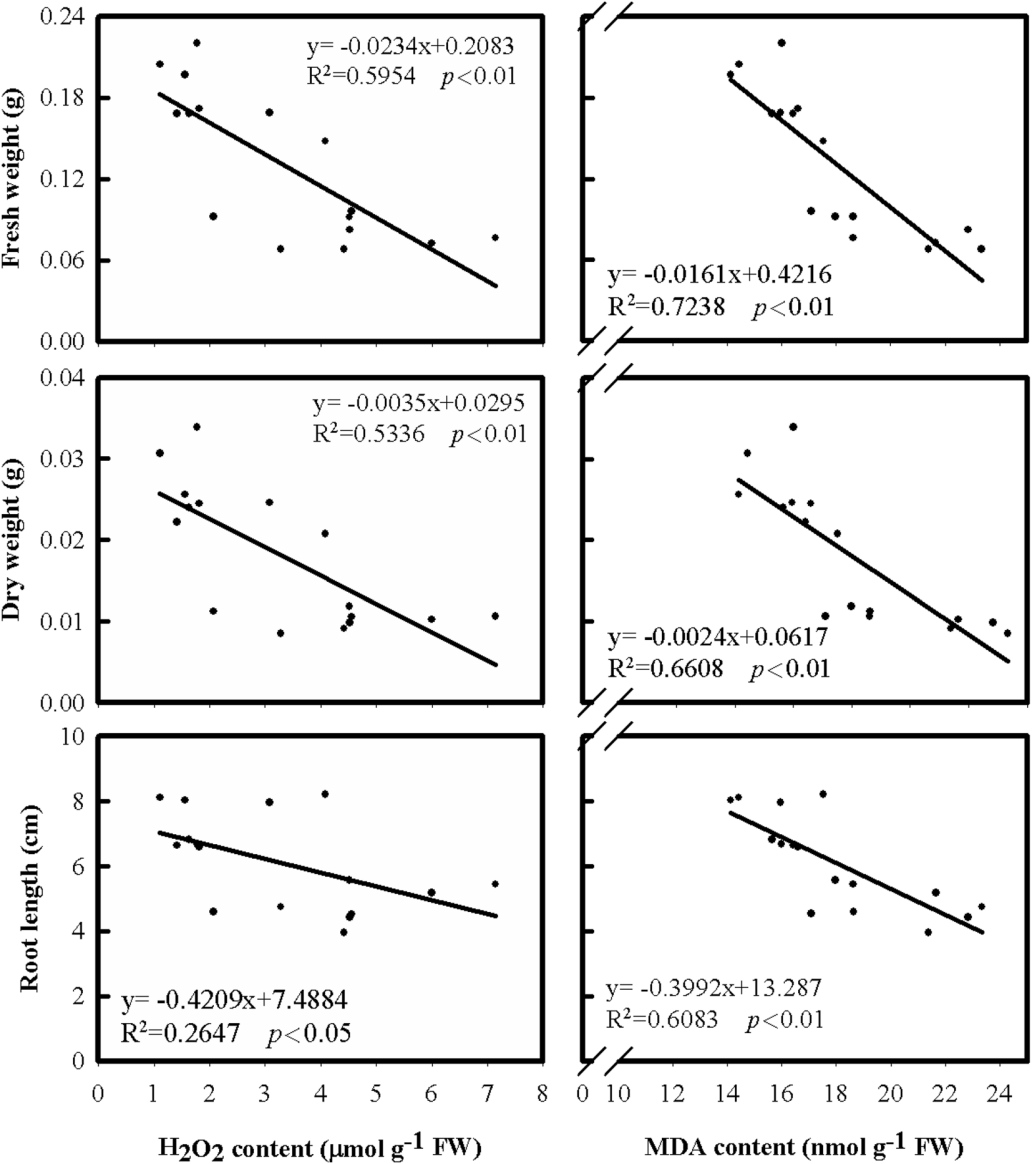
The relationships of root H_2_O_2_ and MDA contents with root fresh weight, dry weight and length under cold treatment among eight rice cultivates. The temperature was set at 15 °C for cold treatment and 27 °C for control. After 4 days of treatment, the temperature was adjusted to 27 °C for 3 days before harvesting

**Fig. 5:**
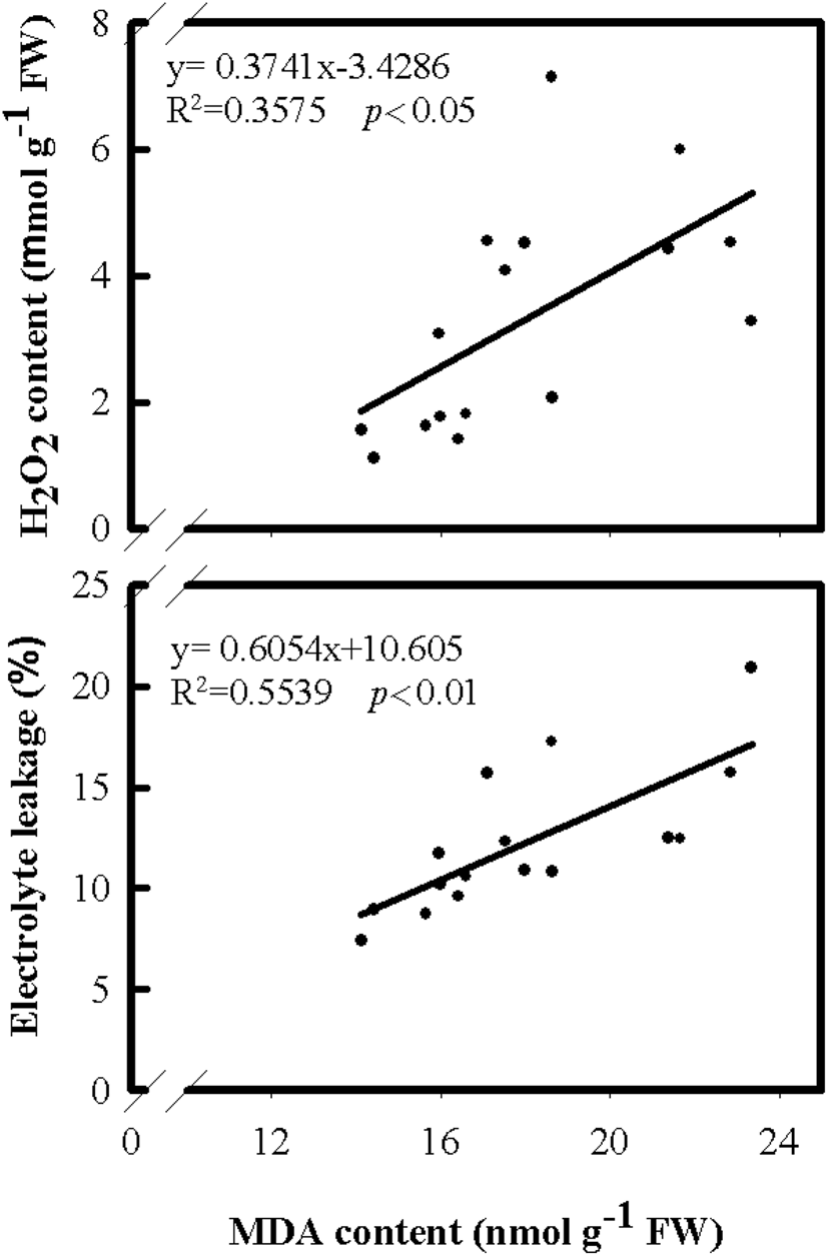
The relationships of root MDA contents with rice root H_2_O_2_ contents and rate of electrolyte leakage under cold treatment among eight cultivars. The temperature was set at 15 °C for cold treatment and 27 °C for control. After 4 days of treatment, the temperature was adjusted to 27 °C for 3 days before harvesting

Since the varieties tested in this study had different extents in response to cold stress, the regression analysis was performed and seeking cold stress-related biochemical traits. The biochemical traits for analysis included the contents of H_2_O_2_ and MDA, and the activities of four antioxidant enzymes, SOD, CAT, APX, and GR. The contents of H_2_O_2_ and MDA were monitored in both shoots and roots of rice after cold treatment (Figs. 3 and 4). The results of this study revealed that the H_2_O_2_ content in shoots was positively correlated with shoot growth (Fig. 3). In contrast, the H_2_O_2_ content in roots was negatively correlated with root growth (Fig. 4), suggesting that the increase of H_2_O_2_ content in shoots might favor the growth of shoot, but the increase of H_2_O_2_ content in roots might reduce the growth of roots. Furthermore, the analysis of MDA contents (Fig. 3), an indicator of cell damage, showed that the MDA contents in shoots only had a weak correlation with shoot growth. Thus, the fluctuation of the H_2_O_2_ contents in shoots under cold treatment found in this study might not be related to the damage of the cells in rice shoots. In contrast, the MDA contents in roots, as well as H_2_O_2_ contents, were negatively correlated with root growth (Fig. 4), suggesting the cells of roots might be damaged by elevated H_2_O_2_ content. Moreover, an increase in electrolyte leakage in roots was found when the MDA content increased (Fig. 5) revealing that the cell damage could occur in the roots under cold treatment.

### Cold stress induces the change of the antioxidant enzymes on the root

**Fig. 6:**
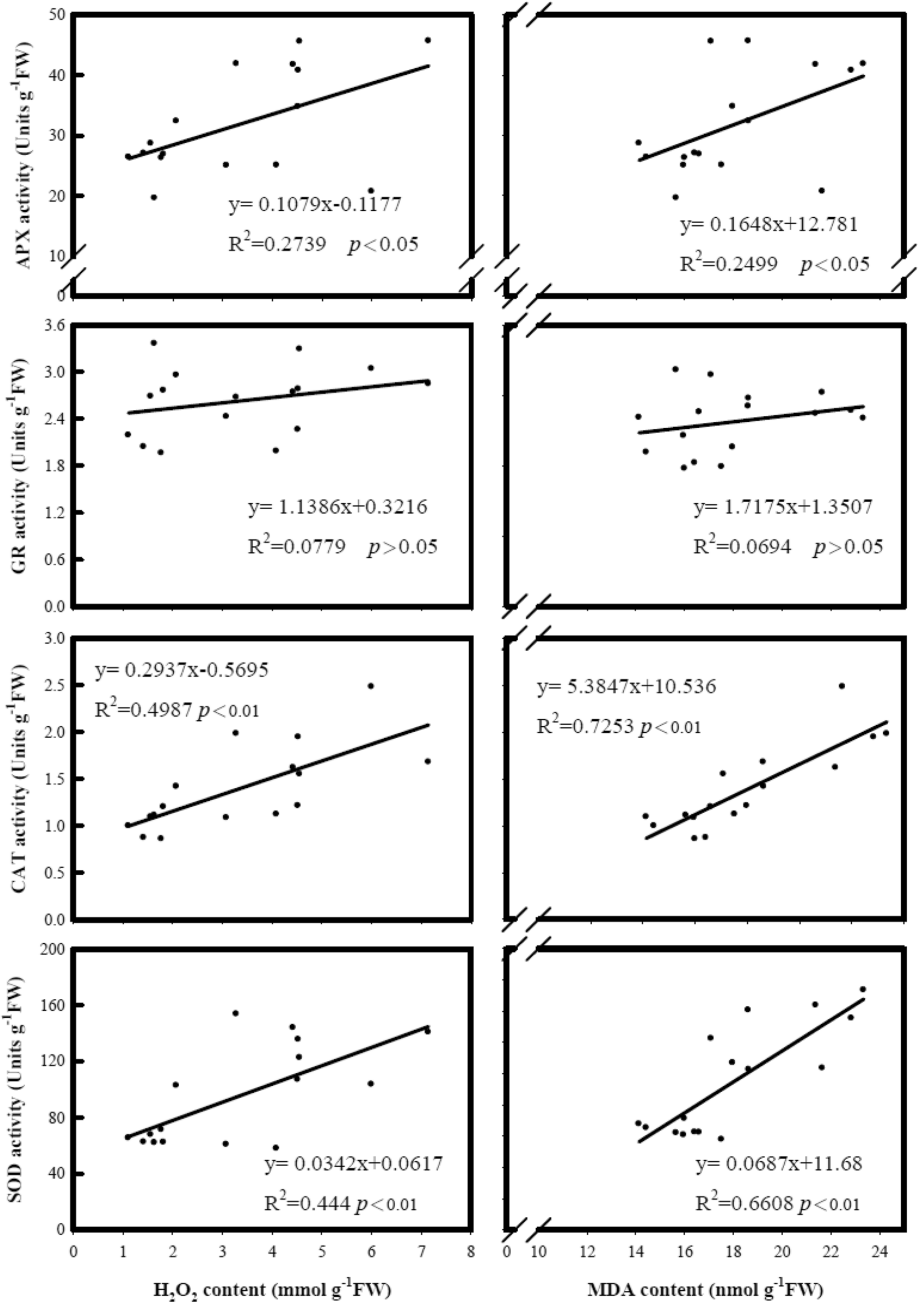
The relationships of root H_2_O_2_ and MDA contents with root superoxide dismutase (SOD), catalase (CAT), ascorbic peroxidase (APX), glutathione reductase (GR) activities under cold treatment among eight cultivars. The temperature was set at 15 °C for cold treatment and 27 °C for control. After 4 days of treatment, the temperature was adjusted to 27 °C for 3 days before harvesting

In order to reveal the possible mechanisms on the regulation of the H_2_O_2_ content in rice roots under cold treatment, this study analyzed the activities of four antioxidant enzymes (Fig. 6), SOD, CAT, APX, and GR. The results of Fig. 6 showed the activities of SOD and CAT correlated well with the increases of H_2_O_2_ and MDA content. However, the activities of APX and GR had a weaker correlation with the increases in H_2_O_2_ and MDA content as compared with that of SOD and CAT.

## Discussion

The reduction of rice yield in subtropical areas due to cold stress is getting attention. This study showed, even with a high portion of temporal region genome, *Japonica* type, most of the productive rice cultivars have the cost-loss with cold damage as high as 75% of all disaster in the subtropical region. Since heat adaptation and tasty were more concerned in previous rice breeding project, the establishment of cold stress-related biochemical traits would be valuable for future rice breeding. In general, most of the current studies on cold stress emphasis the responses of rice shoots to cold treatments. Indeed, this studies showed cold stress induced a high level of H_2_O_2_ content in the shoots of cultivars with higher growth rate (Fig. 3); however, their MDA content was not affected, suggesting the absence of oxidative stress in shoots. The other studies were shown similar results (Morsy et al. 2006; Bonnecarrère et al. 2011). The results of Table 3 showed a decrease in protein content in all tested cultivars after cold treatment. Additionally, the results of the regression analysis (Fig. 2) revealed that the protein content was positively correlated with shoot growth including fresh weight, dry weight, and length, suggesting the more of protein content, the more of shoot growth. There were certain reports showed similar results to our results (Setter and Greenway 1988; Knox 2008; Burton et al. 2010; Neilson et al. 2013; Sampathkumar et al. 2014). Thus, the results above agree with the founding that the reduction of protein turnover under cold treatment could be a result of shoot growth inhibition and leaf development retarding (Setter and Greenway 1988; Yan et al. 2006; Neilson et al. 2013). On the contrary, the results in Table 3 showed that the chlorophyll contents were not affected in most cultivars. In addition, the results of the regression analysis (Fig. 2) revealed that the chlorophyll contents were not correlated with shoot growth among all tested cultivars. Therefore, the results of the above suggested that the rice chlorophyll content was less sensitive to cold stress than the protein content (Table 3). Nevertheless, it should be considered that if cold stress is prolonged, the decrease in chlorophyll could be found as well as the inhibition of shoot growth, that has been reported in many documents. Thus, what the treatment of old temperature at 15 °C had initiated in this study could be just the early responses of rice seedling to cold stress.

The learning of the responses of rice roots to cold stress should not be omitted, accordingly. In fact, the location of the rice root is near the soil surface at the stage of the seedling. At that stage, both rice shoot and root could be facing the same cold temperature at night time. Neilson et al. (2013) indicate when the roots of rice seedlings were exposed to low temperatures, water absorption, and nutrients uptake were reduced from the soil through the root. The results of this study showed that the growth of both shoots and roots decreased in all testing cultivars as compared with the control in regarding the fresh weight, dry weight, and length (Tables 1, 2). It could be considered that the inhibition of root growth under cold stress might be related to water uptake, but not nutrients. Because the materials we used were 8 days-old seedlings could consume nutrients from seeds.

Previous literature showed that salt-induced H_2_O_2_ generation resulted in an increase in ionically bound cell-wall peroxidase activity and followed with the induction of cell-wall stiffening process and the inhibition of root growth (Lin and Kao 2001a, b). Furthermore, a similar result of H_2_O_2_ accumulation was found along with Cd-inhibited root growth of rice seedlings (Cho et al. 2012). The Cd-induced H_2_O_2_ accumulation was responsible for the inhibition of CAT activity (Cho et al. 2012). The results of this research showed an accumulation of H_2_O_2_ content in rice shoots under cold stress (Fig. 3). However, the indication of oxidative stress was found in rice roots only (Figs. 3 and 4). It is likely that the decrease in shoot growth under cold treatment could be a result of the damage on roots, which reduced transportation of water and nutrients to shoots. Moreover, an increase in electrolyte leakage was found in roots under cold treatment (Fig. 5). Thus, the involvement of oxidative stress in rice roots could be the key role of causing cold damage, especially the generation of H_2_O_2_. Therefore, the H_2_O_2_ content decreased in roots under cold treatment can be one of the factors associated with cold-stress tolerance in rice seedlings and could be used for biochemical markers in improving cold tolerance of rice seedling.

The elevation of ROS, such as superoxide anion, singlet oxygen, and H_2_O_2_, has been found in the plant under cold stress (Kuk et al. 2003; Hung et al. 2008; Bhattacharjee 2013) and associated with an increase in lipid peroxidation and damage of the plasma membrane. As we knew, in responding to oxidative stress, plant tissue will increase the activity of SOD to reduce the ROS level and generate H_2_O_2_ (Conklin and Barth 2004; Zhang et al. 2010; Faize et al. 2011, Diaz-Vivancos et al. 2013). Since H_2_O_2_ is toxic to the cell, the activities of CAT or APX will be strengthened to decrease H_2_O_2_ contents (Chou et al. 2012; Chao et al. 2010). Besides, Lin et al. (2016) have shown that the increase of SOD and CAT activity could accelerate ROS reduction. However, our result showed the SOD and CAT activities were positively correlated with the increases of H_2_O_2_ and MDA content (Fig. 6), suggesting the increase of SOD activities promoted H_2_O_2_ accumulation, and the increase of CAT activity was not enough to catalyzed H_2_O_2_. Nevertheless, if CAT or SOD enzyme activities can be enhanced more and able to reduce H_2_O_2_ and MDA content, their role in reducing damage under cold stress could not be ruled out. In summary, lower H_2_O_2_ and MDA contents along with lower SOD activity in rice root could be subjected for rice cold tolerant breeding. Certain cultivars with stronger CAT or SOD activities merit further cold testing.

## Additional file

**Additional file 1: Figure S1.** The average monthly loss of rice yield in Taiwan with different climate disaster during 1999–2009. The monthly loss is expressed by the estimation of the cost of money loss (data from Yao and Chen 2009). Arrows are indicating the occurrence of different climate disaster. **Table S1.** The genetic background of selected rice cultivars for cold treatment.

## Data Availability

All data generated during this study are included in this published article and its additional information file.

## Abbreviations

APX: ascorbic peroxidase
CAT: catalase
GR: glutathione reductase
H_2_O_2_: hydrogen peroxide
MDA: malondialdehyde
SOD: superoxide dismutase
