# Supplementary material for: Biochemical responses of rice roots to cold stress
Source: Bot Stud. 2019 Jul 12;60:14. doi: 10.1186/s40529-019-0262-1 (PMC6626088; doi:10.1186/s40529-019-0262-1)
Supplement: Supplementary file 1 — Additional file 1: Figure S1. The average monthly loss of rice yield in Taiwan with different climate disaster during 1999–2009. The monthly loss is expressed by the estimation of the cost of money loss (data from Yao and Chen 2009). Arrows are indicating the occurrence of different climate disaster. Table S1. The genetic background of selected rice cultivars for cold treatment. [file 40529_2019_262_MOESM1_ESM.docx]

Table S1. The genetic background of selected rice cultivars for cold treatment.

| Genotype | Parents  (male / female) | % of original genome  (*Japonica* / *Indica*) |
| --- | --- | --- |
| Taitung 30  (TT30) | Tai-Keng 6  / Tai-Keng Yu 5025 | 93.75 / 6.25 |
|  |  |  |
| Tainan 11  (TN11) | Tai-Keng Yu 69223  / Tai-Keng 16 | 100 / 0 |
|  |  |  |
| Tainung 71  (TNG71) | Kinuhikari  / Tai-Keng 4 | 93.75 / 6.25 |
|  |  |  |
| Tai-Keng 9  (TK9) | Hokuriku 100  / Tainung-Sen Yu 2414 | 50 / 50 |
|  |  |  |
| Kaohsiung 139  (KH139) | Tainan 5  / Chianung 242 | 100 / 0 |
|  |  |  |
| Tai-Keng 16  (TK16) | Tai-Keng 2  / Tainung 67 | 93.75 / 6.25 |
|  |  |  |
| Tai-Keng 14  (TK14) | Tai-Keng Yu 2011  / Taichung Yu 418 | 87.5 / 12.5 |
|  |  |  |
| Taichung-Sen 10  (TCS10) | Tainung-Sen Yu 204 / Chianung Yu 204 | 0 / 100 |
|  |  |  |


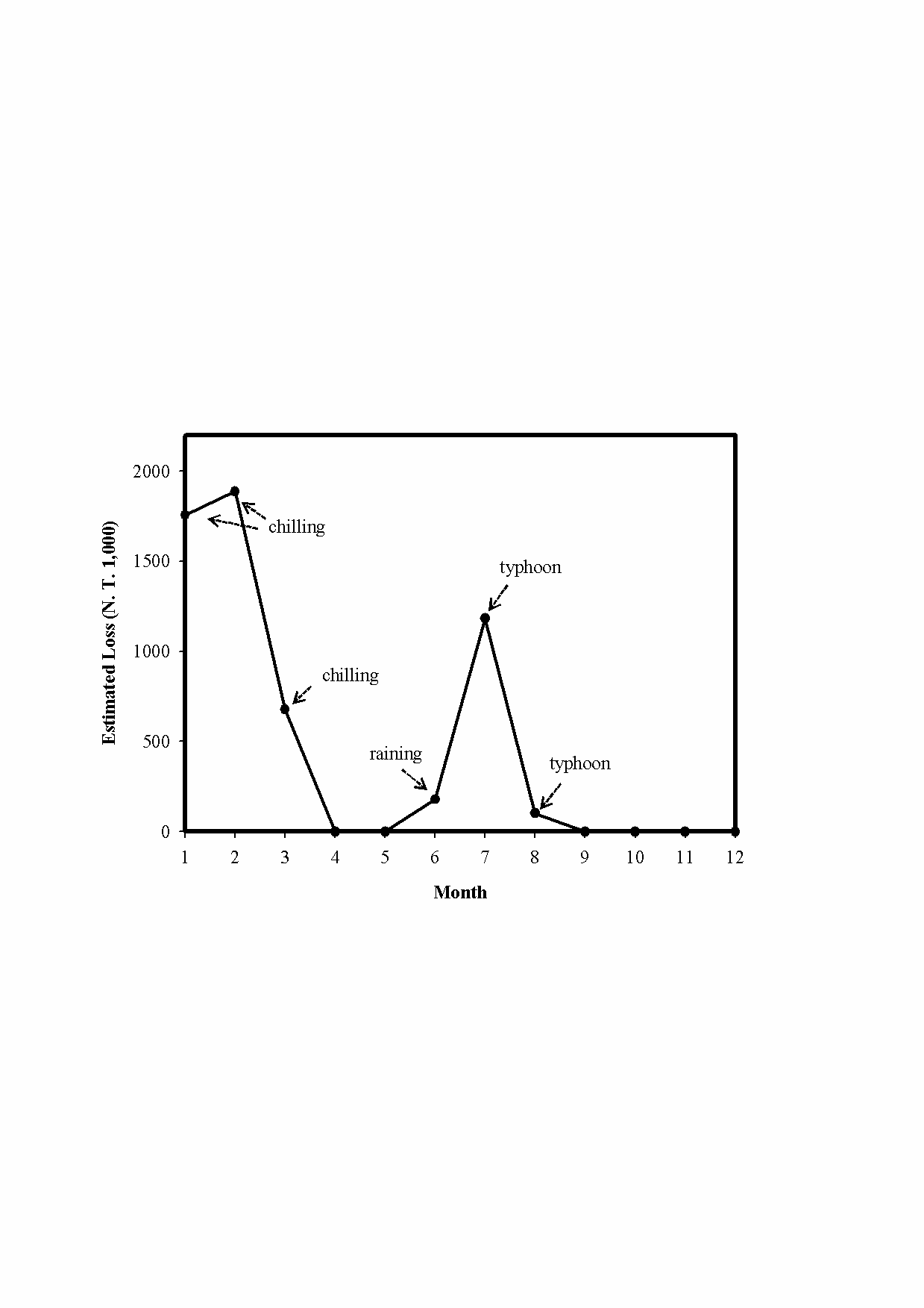


Figure 1s. The average monthly loss of rice yield in Taiwan with different climate disaster during 1999-2009. The monthly loss is expressed by the estimation of the cost of money loss (data from Yao and Chen 2009). Arrows are indicating the occurrence of different climate disaster.

Yao MH and Chen SH (2009) The impact evaluation of climate change on the growth and yield of rice. Crop Enviro Bioinform 6:141-156.
